# Continuous infusion or subcutaneous injection of granulocyte-macrophage colony-stimulating factor: increased efficacy and reduced toxicity when given subcutaneously.

**DOI:** 10.1038/bjc.1996.502

**Published:** 1996-10

**Authors:** A. H. Honkoop, K. Hoekman, J. Wagstaff, C. J. van Groeningen, J. B. Vermorken, E. Boven, H. M. Pinedo

**Affiliations:** Department of Medical Oncology, University Hospital Vrije Universiteit Amsterdam, Netherlands.

## Abstract

Granulocyte-macrophage colony-stimulating factor (GM-CSF) is a haematopoietic growth factor with a wide variety of applications in the clinic. In early phase I studies the continuous intravenous (c.i.) route of administration was often used. Later it was shown that subcutaneous (s.c.) administration was also effective. The optimal route of administration remains, however, poorly defined, and no studies have made a direct comparison between these two routes of administration. We treated patients with advanced breast cancer with moderately high-dose doxorubicin and cylophosphamide and GM-CSF. The first 14 patients received GM-CSF by c.i, while subsequently 47 patients received it s.c. Comparison between the two groups showed that c.i. GM-CSF was more toxic in several respects. There was a higher need for erythrocyte and platelet transfusions and a significant deterioration in the performance status. This study indicates that subcutaneous GM-CSF is the preferred route of administration. Randomised trials are, however, needed to confirm these conclusions.


British Journal of Cancer (1996) 74, 1132-1136
© 1996 Stockton Press All rights reserved 0007-0920/96 $12.00

### Continuous infusion or subcutaneous injection of granulocyte-macrophage colony-stimulating factor: increased efficacy and reduced toxicity when given subcutaneously

AH Honkoop, K Hoekman, J Wagstaff, CJ van Groeningen, JB Vermorken, E Boven and HM Pinedo

*Department of Medical Oncology, University Hospital Vrije Universiteit Amsterdam, Netherlands.*

**Summary** Granulocyte-macrophage colony-stimulating factor (GM-CSF) is a haematopoietic growth factor with a wide variety of applications in the clinic. In early phase I studies the continuous intravenous (c.i.) route of administration was often used. Later it was shown that subcutaneous (s.c.) administration was also effective. The optimal route of administration remains, however, poorly defined, and no studies have made a direct comparison between these two routes of administration. We treated patients with advanced breast cancer with moderately high-dose doxorubicin and cylophosphamide and GM-CSF. The first 14 patients received GM-CSF by c.i, while subsequently 47 patients received it s.c. Comparison between the two groups showed that c.i. GM-CSF was more toxic in several respects. There was a higher need for erythrocyte and platelet transfusions and a significant deterioration in the performance status. This study indicates that subcutaneous GM-CSF is the preferred route of administration. Randomised trials are, however, needed to confirm these conclusions.

**Keywords:** granulocyte-macrophage colony-stimulating factor; route of administration; breast cancer; chemotherapy

Granulocyte-macrophage colony-stimulating factor (GM-CSF) is a haematopoietic growth factor that stimulates the proliferation, maturation and functional properties of neutrophils, monocytes/macrophages and eosinophils (Ruef, 1990). A wide variety of therapeutic applications have evolved for this cytokine. It facilitates haematopoietic recovery after cytotoxic therapy (Harmenberg, 1994), and some trials also showed a reduction in the incidence of neutropenic fever (Gerhartz *et al.*, 1993; Kaplan *et al.*, 1991). In the setting of bone marrow transplantation or peripheral blood progenitor cell transplantation GM-CSF is used either to shorten the time to engraftment or for the mobilisation of peripheral blood progenitor cells (Nemunaitis *et al.*, 1991; Gianni *et al.*, 1989).

Several phase I studies showed that the biological activity of GM-CSF in man is clearly dose dependent and that effective doses are in the range of 1 to 20 $\mu g\ kg^{-1}\ day^{-1}$ (Brandt *et al.*, 1988; Antman *et al.*, 1988; Groopman *et al.*, 1987; Steward *et al.*, 1989; Vadhan-Raj *et al.*, 1987). In these studies GM-CSF was administered by intravenous (i.v.) infusions of different duration, of which the 24 h continuous infusion (c.i.) has been the most widely used. The optimal dose and route of administration, however, remains poorly defined, but certain data suggest that the dose might be in the range of 250 $\mu g\ m^{-2}\ day^{-1}$ (approximately 6 $\mu g\ kg^{-1}\ day^{-1}$) (Edmonson *et al.*, 1989). Lieschke *et al.* (1989) were the first to show that the subcutaneous (s.c.) route of administration at dosages of 3-15 $\mu g\ kg^{-1}$ was also effective at inducing leucocytosis and was tolerated well by the patients.

No studies have made a direct comparison between c.i. and s.c. GM-CSF. Comparison between studies is complicated because different patient populations and different concomitant therapies would be expected to influence the response to GM-CSF. We performed a phase II study in which we treated patients with advanced breast cancer with a dose-intensive regimen of doxorubicin and cyclophosphamide in combination with GM-CSF. Initially a pilot study was done to establish the dose for this regimen (Hoekman *et al.*, 1991*a*). The first patients received c.i. GM-CSF, the subsequent patients received s.c. GM-CSF. Although it was not a randomised trial, the identical chemotherapeutic regimen and patient selection criteria for patients treated with either GM-CSF c.i. or s.c. makes it possible to compare these two routes of administration in terms of toxicity and efficacy.

Correspondence: HM Pinedo, Head of Department of Medical Oncology, University Hospital Vrije Universiteit, PO Box 7057, 1007 MB Amsterdam, The Netherlands
Received 2 January 1996; revised 25 March 1996; accepted 19 April 1996

### Patients and methods

### *Patient selection*

Eligible patients were women between 18 and 65 years of age with locally advanced or metastatic breast cancer and a performance status of 2 or less according to World Health Organization (WHO) criteria. No prior chemotherapy for advanced disease was allowed. Adequate bone marrow function (white blood cell count $\geqslant 4.0 \times 10^9\ l^{-1}$ and platelet count $\geqslant 100 \times 10^9\ l^{-1}$), renal function (serum creatinine $\leqslant 150\ \mu mol\ l^{-1}$) and hepatic function (serum bilirubin $\leqslant 25\ \mu mol\ l^{-1}$) were required. A history of cardiovascular disease and/or a left ventricular ejection fraction (LVEF) <50% were exclusion criteria. All patients gave written informed consent and the protocol was approved by the ethical and scientific review committees of the University Hospital Vrije Universiteit Amsterdam.

### *Treatment*

Treatment consisted of doxorubicin and cyclophosphamide by i.v. bolus injection every 21 days. *E. coli*-derive non-glycosylated recombinant human GM-CSF 250 $\mu g\ m^{-2}$-$day^{-1}$ was started at day 2 and given for 10 days. The first 14 patients received GM-CSF c.i. via an implantable drug delivery system (Port-A-Cath, Pharmacia Deltec, St Paul, USA) and a portable infusion pump (CADD-1, Pharmacia Deltec). In the subsequent 47 patients GM-CSF was given s.c. The first six patients (group A) with c.i. GM-CSF received it from the second cycle onwards. They had a dose reduction of doxorubicin and cyclophosphamide in cycles 3 and 5. The remaining eight patients with c.i. GM-CSF (group B) and the 47 patients with s.c. GM-CSF (group C) received GM-CSF from the first cycle onwards. They had a dose reduction of doxorubicin and cyclophosphamide in cycles 2 and 4. It was the intention to treat patients with six cycles, but when a complete remission was reached earlier only one extra cycle was given as consolidation. Treatment was delayed for 1 week if the neutrophil count was $<2 \times 10^9\ l^{-1}$, platelets were $<100 \times 10^9\ l^{-1}$, or in the presence of active infection, mucositis or if the performance status had deteriorated to WHO grade 3 or 4. Erythrocyte transfusions were given when the haemoglobin level decreased to $<6.0$ mmol $l^{-1}$, and prophylactic platelet transfusions were given when the platelet count was $<10 \times 10^9\ l^{-1}$, or at higher counts when evidence of bleeding was observed. When there was a decline in LVEF below 50% chemotherapy was discontinued. Fever was judged to be neutropenic fever requiring intravenous antibiotic treatment if axillary body temperature was 38.5°C lasting more than 4 h, and neutrophils were below $0.5 \times 10^9\ l^{-1}$.

### *Clinical and laboratory monitoring*

While on treatment, all patients had a medical history, physical examination, baseline laboratory tests, chest radiograph and ECG before each cycle. Patients had a physical examination weekly. Between cycles they were asked to record their axillary temperature twice daily and to note any specific complaints. Full blood counts, including differential cell counts, were performed three times a week. Biochemical analysis was carried out weekly. LVEF was performed every second cycle and before the sixth cycle. Tumour response was evaluated before each cycle for patients with locally advanced

**Table I** Patient characteristics

| | *c.i. GM-CSF* | *s.c. GM-CSF* |
|---|---|---|
| Total number of patients | 14 | 47 |
| Locally advanced breast cancer | 4 | 29 |
| Metastatic breast cancer | 10 | 18 |
| Age, median (range) | 48 (37–60) | 47 (28–65) |
| Performance status (WHO) | | |
| 0 | 12 | 43 |
| 1 | 2 | 4 |
| 2 | 0 | 0 |
| Prior adjuvant chemotherapy | 0 | 0 |
| Metastatic sites involved | | |
| Breast | 3 | 5 |
| Lung | 6 | 7 |
| Liver | 4 | 8 |
| Nodes | 5 | 5 |
| Skin | 2 | 3 |
| Bone | 4 | 9 |
| Bone marrow | | |
| Number of cycles median (range) | 4.5 (2–6) | 5 (1–6) |
| Dose intensity median (range) (mg $m^{-2}$ per week) | | |
| Doxorubicin | 27 (23–29) | 27 (23–30) |
| Cyclophosphamide | 281 (238–319) | 278 (236–330) |
| Total dose, median (range) (mg $m^{-2}$) | | |
| Doxorubicin | 368 (180–495) | 405 (90–480) |
| Cyclophosphamide | 3875 (2000–3980) | 4250 (1000–5000) |

**Table II** Nadirs (median, range) and duration of neutropenia and thrombocytopenia ($\times 10^9\ l^{-1}$) and erythrocyte and platelet transfusions with sequential cycles of chemotherapy among the different treatment groups

| *Group and cycle no.* | *Doses of doxorubicin cyclophosphamide (mg $m^{-2}$)* | *No. of patients* | *Neutrophil count Nadir (range)* | *Neutrophil count Days<0.5 (range)* | *Platelet count Nadir (range)* | *Platelet count Days<50 (range)* | *No. of cycles with erythrocyte transfusions* | *No. of cycles with platelet transfusion* |
|---|---|---|---|---|---|---|---|---|
| Group A ($n=6$) | | | | | | | | |
| 1 | 90/1000 | 6 | 0.02 (0.01–0.03) | 7 (3–9) | 65 (58–70) | 0 (0–0) | 0 | 0 |
| 2[a] | 90/1000 | 6 | 0.19 (0.00–0.22) | 6 (5–7) | 48 (17–62) | 3 (0–5) | 2 | 0 |
| 3[a] | 82.5/875 | 5 | 0.14 (0.00–0.34) | 7 (3–7) | 17 (6–33) | 7 (5–9) | 4 | 1 |
| 4[a] | 82.5/875 | 3 | 0.08 (0.00–0.24) | 5 (4–9) | 16 (6–28) | 12 (7–12) | 3 | 0 |
| 5[a] | 75/750 | 3 | 0.02 (0.04–0.22) | 7 (6–9) | 11 (7–16) | 9 (5–9) | 3 | 2 |
| 6[a] | 75/750 | 2 | 0.02 (0.00–0.04) | 6 (5–6) | 9 (9–9) | 10 (9–11) | 2 | 2 |
| Group B ($n=8$) | | | | | | | | |
| 1[a] | 90/1000 | 8 | 0.09 (0.00–0.36) | 4 (3–7) | 72 (54–119) | 0 (0–5) | 3 | 0 |
| 2[a] | 82.5/875 | 8 | 0.20 (0.06–0.60) | 2 (0–7) | 54 (16–92) | 0 (0–7) | 2 | 0 |
| 3[a] | 82.5/875 | 8 | 0.13 (0.00–0.22) | 5 (4–8) | 28 (11–58) | 6 (0–9) | 8 | 0 |
| 4[a] | 75/750 | 8 | 0.16 (0.00–0.18) | 6 (4–9) | 30 (3–76) | 4 (0–9) | 8 | 3 |
| 5[a] | 75/750 | 4 | 0.09 (0.00–0.27) | 6 (6–6) | 14 (6–71) | 7 (7–14) | 4 | 2 |
| 6[a] | 75/750 | 3 | 0.04 (0.00–0.21) | 3 (0–6) | 6 (6–15) | 9 (9–11) | 2 | 2 |
| Group C ($n=47$) | | | | | | | | |
| 1[b] | 90/1000 | 47 | 0.04 (0.00–0.08) | 6 (0–8) | 109 (17–238) | 0 (0–11) | 4 | 3 |
| 2[b] | 82.5/875 | 45 | 0.18 (0.00–0.24) | 7 (0–9) | 169 (16–224) | 0 (0–7) | 10 | 0 |
| 3[b] | 82.5/875 | 45 | 0.05 (0.00–0.38) | 6 (0–9) | 99 (7–108) | 0 (0–9) | 15 | 4 |
| 4[b] | 75/750 | 42 | 0.04 (0.00–0.24) | 6 (0–9) | 57 (5–142) | 0 (0–10) | 21 | 5 |
| 5[b] | 75/750 | 34 | 0.05 (0.00–0.18) | 6 (0–9) | 35 (4–116) | 3 (0–7) | 19 | 7 |
| 6[b] | 75/750 | 21 | 0.06 (0.00–0.11) | 5 (0–9) | 17 (3–65) | 5 (0–10) | 11 | 5 |

[a]Chemotherapy cycles with GM-CSF c.i. [b]Chemotherapy with GM-CSF s.c.

breast cancer and every second cycle for patients with metastatic breast cancer. Response and toxicities were scored according to WHO criteria.

*Statistical analysis*

For toxicity as well as for erythrocyte and platelet transfusion, the percentage of patients with c.i. GM-CSF with a certain event were compared with the percentage of patients with s.c. GM-CSF with a certain event. Furthermore, all cycles with c.i. GM-CSF were compared with all cycles with s.c. GM-CSF. Differences were assessed by Fisher's exact test (two-tail).

### Results

The characteristics of the 61 entered patients, the number of chemotherapy cycles, the dose intensity and total dose of chemotherapy in the different treatment groups are depicted in Table I. Two groups of patients who received c.i. GM-CSF (group A and group B) and the group of patients who received s.c. GM-CSF (group C) were available for analysis of the effects of GM-CSF. Data on the comparison between cycles without and with GM-CSF have been published elsewhere (Hoekman *et al.*, 1991*a*).

Table II shows the treatment regimens and the median nadir of neutrophils and platelets and the median duration of neutropenia and thrombocytopenia in the different groups per cycle. There was no difference in neutrophil nadir or neutrophil recovery to a value of $\geqslant 2 \times 10^9 l^{-1}$ (Table I, Figure 1). For every cycle mean platelet values were lower in the c.i group. This difference became greater as more cycles were given (Table I, Figure 2). Platelet counts $< 10 \times 10^9 l^{-1}$ were observed on 11 and 22 occasions during 58 c.i. cycles and 256 s.c. cycles respectively ($P = 0.04$).

Erythrocyte transfusions and platelet transfusions were given mainly in later cycles (Table II). Erythrocyte transfusions were necessary in all patients with c.i. GM-CSF *vs* 33/47 (70%) patients with s.c. GM-CSF ($P = 0.02$), and in 41/58 (70%) cycles with c.i. GM-CSF *vs* 80/256 (31%) cycles with s.c. GM-CSF ($P < 0.0001$). Platelet transfusions were given in 10/14 (71%) patients with c.i. GM-CSF *vs* 20/47 (43%) patients with s.c. GM-CSF ($P = 0.07$), and in 12/58 (21%) cycles with c.i. GM-CSF *vs* 24/256 (9%) cycles with s.c. GM-CSF ($P = 0.02$).

Comparative data for non-haematological toxicity are shown in Table III. Hospital admission because of toxicity was necessary in 10/14 (71%) c.i. GM-CSF patients *vs* 20/47 (43%) s.c. GM-CSF patients ($P = 0.03$), and in 20/58 (34%) c.i. cycles *vs* 38/256 (15%) s.c. cycles ($P = 0.0005$). Despite this, there was no difference in treatment delay or discontinuation of therapy because of toxicity between the c.i. and s.c. GM-CSF groups. Treatment delay occurred in 6/14 (42%) c.i. patients *vs* 20/47 (43%) s.c. patients ($P > 0.9$), and in 8/58 (14%) c.i. GM-CSF cycles *vs* 27/256 (11%) s.c. cycles ($P = 0.49$). Treatment was discontinued in 4/14 (29%) c.i. GM-CSF patients *vs* 7/47 (15%) s.c. GM-CSF patients ($P = 0.25$).

Response rates were comparable. Twelve out of 14 (86%) patients in the c.i. GM-CSF group and 43/47 (91%) patient in the s.c. GM-CSF group showed a complete or partial response ($P = 0.61$).

### Discussion

This study, although not randomised, indicates that c.i. GM-CSF is more toxic in several respects compared with s.c. GM-CSF. Erythrocyte and platelet transfusions were needed more often with c.i. GM-CSF and there was a significant difference in deterioration of the performance status of the patients.

Both routes of administration of GM-CSF were equally effective as far as recovery of neutrophils is concerned. Mean

**Figure 1** Neutrophil counts of patients treated with doxorubicin and cyclophosphamide, either with c.i. GM-CSF (□) or s.c. GM-CSF (○). Values are given as means and s.d.

**Figure 2** Platelet counts of patients treated with doxorubicin and cyclophosphamide, either with c.i. GM-CSF (□) or s.c. GM-CSF (○). Values are given as means and s.d.

Table III Comparison of toxicity between c.i. GM-CSF and s.c. GM-CSF

| | Analysis per patient | | | Analysis per cycle | | |
|---|---|---|---|---|---|---|
| | c.i. GM-CSF | s.c. GM-CSF | P-value | c.i. GM-CSF | s.c. GM-CSF | P-value |
| Total number | 14 | 47 | | 58 | 256 | |
| Toxicity | | | | | | |
| Neutropenic fever with antibiotics | 9 | 14 | 0.03 | 13 | 25 | 0.01 |
| Positive blood culture | 3 | 4 | 0.4 | 4 | 5 | 0.06 |
| Performance status > 2 | 6 | 5 | 0.01 | 35 | 25 | <0.0001 |
| Stomatitis ≥ grade 3 | 8 | 8 | 0.005 | 12 | 20 | 0.006 |
| Nausea/vomiting ≥ grade 3 | 3 | 5 | 0.4 | 6 | 12 | 0.11 |
| Liver enzyme disturbance grade 2–3 | 3 | 9 | >0.9 | 17 | 63 | 0.5 |
| Cutaneous reactions | – | 18 | – | – | – | – |
| Subclavian vein thrombosis | 3 | – | – | – | – | – |
| Decline in LVEF below 50% | 2 | 6 | 0.3 | – | – | – |

platelet values were lower in the group receiving c.i. GM-CSF, and there was a trend for more platelet transfusions, although this did not reach significance. We did not change the policy of platelet transfusion during the study. The effect of GM-CSF on platelet counts has varied in reported studies from no effects (Brandt *et al.*, 1988; Groopman *et al.*, 1987; Antin *et al.*, 1988) to an increase in recovery of platelets (Vadhan-Raj *et al.*, 1987, 1988). GM-CSF might induce the production of interleukin-6 (De Vries *et al.*, 1991), which can stimulate thrombopoiesis (Hill, 1990). Maybe with the s.c. administration this is more pronounced, because GM-CSF is injected into the skin, where accessory cells bearing GM-CSF receptors are present. These cells could then be responsible for the generation of secondary cytokines, such as interleukin-6, which can in part compensate for the suppression of thrombopoiesis.

Erythrocyte infusions were also required more often in the c.i. GM-CSF group. Indications for blood transfusions did not change during the study. A cumulative anaemia has been previously described during the use of GM-CSF (Ardizzoni *et al.*, 1994; O'Shaugnessy *et al.*, 1994; Suderland, 1991), but this marked difference in transfusion requirement has not been reported before. A probable explanation could be that tumour necrosis factor (TNF), which is an inhibitor of erythropoiesis (Rusten, 1995) and whose production is stimulated by GM-CSF (Sisson, 1988) is released for a prolonged period or in higher concentrations when GM-CSF is administered by the c.i. route.

Neutropenic fever judged to require intravenous antibiotics occurred more often in the group receiving c.i. GM-CSF. A possible explanation can be that our ability to discern whether fever was due to infection or to GM-CSF improved as the study progressed. Another explanation may be the presence of the Port-A-Cath, although the percentage of patients with a positive blood culture was not different between the two groups.

Various other non-haematological side-effects were observed. In three out of 14 patients who received c.i. GM-CSF a subclavian vein thrombosis developed. This complication has been described by others as well (Antman *et al.*, 1988), and probably results from the release of TNF, which is an initiator of the coagulation cascade (Nawroth, 1986). There was a noticeable difference in the incidence of stomatitis which was worse in the c.i. GM-CSF group. This occurred despite the fact that there were no significant differences in neutrophil nadir and duration of neutropenia, which usually parallels the course of stomatitis (Lockhart, 1979). Liver enzyme disturbances were equal in both groups. It seems that this side-effect is correlated with the dose and not with the route of administration of GM-CSF (Cebon *et al.*, 1992). Erythematous reactions at injection sites have been described in several patients using s.c. GM-CSF (Lieschke *et al.*, 1989). In our group it was recorded in 38% of patients. The symptoms could generally be relieved with antihistamines. Earlier we reported on thyroid dysfunction during c.i. GM-CSF treatment in two patients with pre-existing thyroid antibodies (Hoekman *et al.*, 1991*b*). In our subsequent patients with s.c. GM-CSF we did not observe this phenomenon.

General weakness resulting in a decline in performance status was significantly worse in patients receiving c.i. GM-CSF. The reason is unclear. It is known that the side-effects of GM-CSF are in part mediated by the release of secondary cytokines such as TNF and interleukin-6 (De Vries *et al.*, 1991; Stehle *et al.*, 1990). The efficacy of GM-CSF seems to correlate with the duration for which serum levels are maintained above 1 ng $ml^{-1}$ (Cebon *et al.*, 1988). It is not known if this is also true for the side-effects. For s.c. administration serum levels $>1$ ng $ml^{-1}$ are achieved for approximately 16 h (Lieschke *et al.*, 1990). For c.i. administration pharmacokinetics are not well known but when adequate doses are used there may be a continuous level above 1 ng $ml^{-1}$.

GM-CSF administered by c.i. is still used on several occasions (Bishop *et al.*, 1994; Gordon *et al.*, 1994). There are situations where the s.c. administration is not attractive, for instance during prolonged and severe thrombocytopenia, but one should bear in mind that c.i. GM-CSF is accompanied by more side-effects. In our study there was no significant difference in treatment delay or in discontinuation of therapy between patients receiving c.i. GM-CSF or s.c. GM-CSF. One can imagine, however, that with such a difference in toxicity profile c.i. GM-CSF might have a negative effect on the delivered chemotherapy dose intensity, which seems to be an important determinant of the outcome in several clinical situations.

This study indicates that the s.c. route of administration of GM-CSF is to be preferred over the c.i. route. These results warrant further study in a randomised trial.

### References

ANTIN SJ, SMITH BR, HOLMES W AND ROSENTHAL DS. (1988). Phase I/II study of recombinant human granulocyte–macrophage colony-stimulating factor in aplastic anemia and myelodysplastic syndrome. *Blood*, **72,** 705–713.

ANTMAN K, GRIFFIN J, ELIAS A, SOCINKSI MA, RYAN L, CANNISTRA SA, OETTE D, WHITLEY M, FREI III E AND SCHNIPPER LE. (1988). Effects of recombinant human granulocyte–macrophage colony-stimulating factor on chemotherapy-induced myelosuppression. *N. Engl. J. Med.*, **319,** 593–598.

ARDIZZONI A, VENTURINI M, SERTOLI MR, GIANNESSI PG, BREMA F, DANOVA M, TESTORE F, MARIANA GL, PENNUCCI MC, OUEIROLO P, SILVESTRO S, BRUZZI P, LIONETTO R, LATINI F AND ROSSO R. (1994). Granulocyte-macrophage colony-stimulating factor (GM-CSF) allows acceleration and dose intensity increase of CEF chemotherapy: a randomised study in patients with advanced breast cancer. *Br. J. Cancer*, **69,** 385–391.

BISHOP MR, ANDERSON JR, JACKSON JD, BIERMAN PJ, REED EC, VOSE JM, ARMITAGE JO, WARKENTIN PI AND KESINGER A. (1994). High dose therapy and peripheral blood progenitor cell transplantation: effects of recombinant human granulocyte-macrophage colony-stimulating factor on the autograft. *Blood*, **83**, 610-616.

BRANDT SJ, PETERS WP, ATWATER SK, KURTZENBERG J, BOROWITZ MJ, JONES RB, SHPALL EJ, BAST RC, GILBERT CJ AND OETTE DH. (1988). Effect of recombinant granulocyte-macrophage colony-stimulating factor on hematopoietic reconstitution after high-dose chemotherapy and autologous bone marrow transplantation. *N. Engl. J. Med.*, **318**, 869-876.

CEBON J, DEMPSEY P, FOX RM, KANNOURAKIS G, BONNEM E, BURGESS AW AND MORSTYN G. (1988). Pharmacokinetics of human granulocyte-macrophage-stimulating factor (hGM-CSF) using a sensitive immunoassay. *Blood*, **72**, 1340-1347.

CEBON J, LIESCHKE GJ, BURY RW AND MORSTYN G. (1992). The dissociation of GM-CSF efficacy from toxicity according to route of administration: a pharmacodynamic study. *Br. J. Haematol.*, **80**, 144-150.

DE VRIES EGE, WILLEMSE PHB, BIESMA B, STERN AC, LIMBURG PC AND VELLENGA E. (1991). Flare-up of rheumatoid arthritis during GM-CSF treatment after chemotherapy. *Lancet*, **338**, 517-518.

EDMONSON JH, LONG HJ, JEFFRIES JA, BUCKNER JC, COLON-OTERO G AND FITCH TR. (1989). Amelioration of chemotherapy-induced thrombocytopenia by GM-CSF: apparent dose and schedule dependency. *J. Natl Cancer Inst.*, **81**, 1510-1512.

GERHARTZ HH, ENGELHART M, MEUSERS P, BRITTINGER G, WILMANNS W, SCHLIMOK G, MUELLER P, HUHN D, MUSCH R AND SIEGERT W. (1993). Randomized, double-blind, placebo-controlled, phase III study of recombinant human granulocyte-macrophage colony-stimulating factor as adjunct to induction treatment of high-grade non-Hodgkin's lymphomas. *Blood*, **82**, 2329-2339.

GIANNI AM, SIENA S, BREGNI M, TARELLA C, STERN AC, PILERI A AND BONADONNA G. (1989). Granulocyte-macrophage colony-stimulating factor to harvest circulating haemapoietic stem cells for autotransplantation. *Lancet*, **2**, 580-585.

GORDON B, SPADINGER A, HODGES E, RUBY E, STANLY R AND COCCIA P. (1994). Effect of granulocyte-macrophage colony-stimulating factor on oral mucositis after hematopoietic stem-cell transplantation. *J. Clin. Oncol.*, **12**, 1917-1922.

GROOPMAN JE, MITSUYASU RT, DELEO MJ, OETTE DH AND GOLDE DW, (1987). Effect of recombinant human granulocyte-macrophage colony-stimulating factor on myelopoiesis in the acquired immunodeficiency syndrome. *N. Engl. J. Med.*, **317**, 593-598.

HARMENBERG J, HOGLUND M AND HELLSTROM-LINDBERG E. (1994). G- and GM-CSF in oncology and oncological haematology. *Eur. J. Haematol.* (suppl.) **55**, 1-28.

HILL RJ, WARREN MK AND LEVIN J. (1990). Stimulation of thrombopoiesis in mice by human recombinant interleukin 6. *J. Clin. Invest.*, **85**, 1242-1247.

HOEKMAN K, WAGSTAFF J, VAN GROENINGEN CJ, VERMORKEN JB, BOVEN E AND PINEDO HM. (1991*a*). Effects of recombinant human granulocyte-macrophage colony-stimulating factor on myelosuppression induced by multiple cycles of high dose chemotherapy in patients with advanced breast cancer. *J. Natl Cancer Inst.*, **83**, 1546-1553.

HOEKMAN K, VON BLOMBERG-VAN DER FLIER BME, WAGSTAFF J, DREXHAGE HA AND PINEDO HM. (1991*b*). Reversible thyroid dysfunction during treatment with GM-CSF. *Lancet*, **338**, 541-542.

KAPLAN LD, KAHN JO, CROWE S, NORDTFELT D, NEVILLE P, GROSSBERG H, ABRAMS DI, TRACEY J, MILLS J AND VOLBERDING PA. (1991). Clinical and virologic effects of recombinant human granulocyte-macrophage colony-stimulating factor in patients receiving chemotherapy for human immunodeficiency virus associated non-Hodgkin's lymphoma: results of a randomized trial. *J. Clin. Oncol.*, **9**, 929-940.

LIESCHKE GJ, MAHER D, CEBON J, O'CONNER M, GREEN M, SHERIDAN W, BOYD A, RALLINGS M, BONNEM E, METCALF D, BURGESS AW, MCGRATH K, FOX RM AND MORSTYN G. (1989). Bacterially synthesized recombinant human granulocyte colony-stimulating factor in patients with advanced malignancy. *Ann. Intern. Med.*, **110**, 357-364.

LIESCHKE GJ, MAHER D, O'CONNOR M, GREEN M, SHERIDAN W, RALLINGS M, BONNEM E, BURGESS AW, MCGRATH K, FOX RM AND MORSTYN G. (1990). Phase I study of intravenously administered bacterially synthesized granulocyte-macrophage colony-stimulating factor and comparison with subcutaneous administration. *Cancer Res.*, **50**, 606-614.

LOCKHART PB AND SONIS ST. (1979). Relationship of oral complications to peripheral blood leucocyte and platelet counts in patients receiving cancer chemotherapy. *Oral Surg.*, **48**, 21-28.

NAWROTH PP AND STERN DM. (1986). Modulation of endothelial cell hemostatic properties by tumor necrosis factor. *J. Exp. Med.*, **163**, 740-745.

NEMUNAITIS J, RABINOWE SN, SINGER JW, BIERMAN PJ, VOSE JM, FREEDMAN AS, ONETTO N, GILLIS S, OETTE D, GOLD M, BUCKNER D, HANSEN JA, RITZ J, APPELBAUM FR, ARMITAGE JO AND NADLER LM. (1991). Recombinant granulocyte-macrophage stimulating factor after autologous bone marrow transplantation for lymphoid cancer. *N. Engl. J. Med.*, **324**, 1773-1778.

O'SHAUGNESSY JA, DENICOFF AM, VENZON DJ, DANFORTH D, PIERCE LJ, FRAME JN, BASTIAN A, GHOSH B, GOLDSPIEL B, MILLER L, DORR FA, KEEGAN P, BENBARUCH N, MROSE H, NOONE M AND COWAN KH. (1994). A dose intensity study of FLAC (5-fluorouracil, leucovorin, doxorubicin, cyclophosphamide) chemotherapy and *Escherichia coli*-derived granulocyte-macrophage colony-stimulating factor (GM-CSF) in advanced breast cancer patients. *Ann. Oncol.*, **5**, 709-716.

RUEF C AND COLEMAN DL. (1990). Granulocyte-macrophage colony-stimulating factor: pleiotropic cytokine with potential clinical usefulness. *Rev. Infect. Dis.*, **12**, 41-62.

RUSTEN LS AND JACOBSEN SE. (1995). Tumor necrosis factor (TNF)-alpha directly inhibits human erythropoiesis *in vitro*: role of p55 and p75 TNF receptors. *Blood*, **85**, 989-996.

SISSON SD AND DINARELLO CA. (1988). Production of interleukin-1$\alpha$, interleukin-$\beta_1$ and tumor necrosis factor by human nuclear cells stimulated with granulocyte-macrophage colony-stimulating factor. *Blood*, **72**, 1368-1374.

STEHLE B, WEIS C, HO AD AND HUNSTEIN W. (1990). Serum levels of tumor necrosis factor $\alpha$ in patients treated with granulocyte-macrophage colony-stimulating factor. *Blood*, **75**, 1895-1896.

STEWARD WP, SCARFFE JH, AUSTIN R, BONNEM E, THATCHER N, MORGENSTERN G AND CROWTHER D. (1989). Recombinant human granulocyte-macrophage colony-stimulating factor (rhGM-CSF) given as daily short infusion-a phase I dose-toxicity study. *Br. J. Cancer*, **59**, 142-145.

SUDERLAND M, ABELOFF M AND NEIDHART J. (1991). Continuous GM-CSF failed to ameliorate neutropenia and worsened thrombocytopenia in a dose-intensive regimen for breast cancer patients (abstract). *Breast Cancer Res. Treat.*, **19**, 158.

VADHAN-RAJ S, KEATING M, LEMAISTRE A, HITTELMAN WN, MCGREDIE K, TRUJILLO JM, BOXMEYER HE, HENNEY C AND GUTTERMAN JU. (1987). Effects of recombinant human granulocyte-macrophage colony-stimulating factor in patients with myelodysplastic syndromes. *N. Engl. J. Med.*, **317**, 1545-1552.

VADHAN-RAJ S, BEUSCHER S, HORWITZ LJ, LEMAISTRE A, KEATING M, WALTERS W, VENTURA C, HITTELMAN W, BROXMEYER HE AND GUTTERMAN JU. (1988). Stimulation of hematopoiesis in patients with bone marrow failure and in patients with malignancy by recombinant human granulocyte-macrophage colony-stimulating factor. *Blood*, **72**, 134-141.


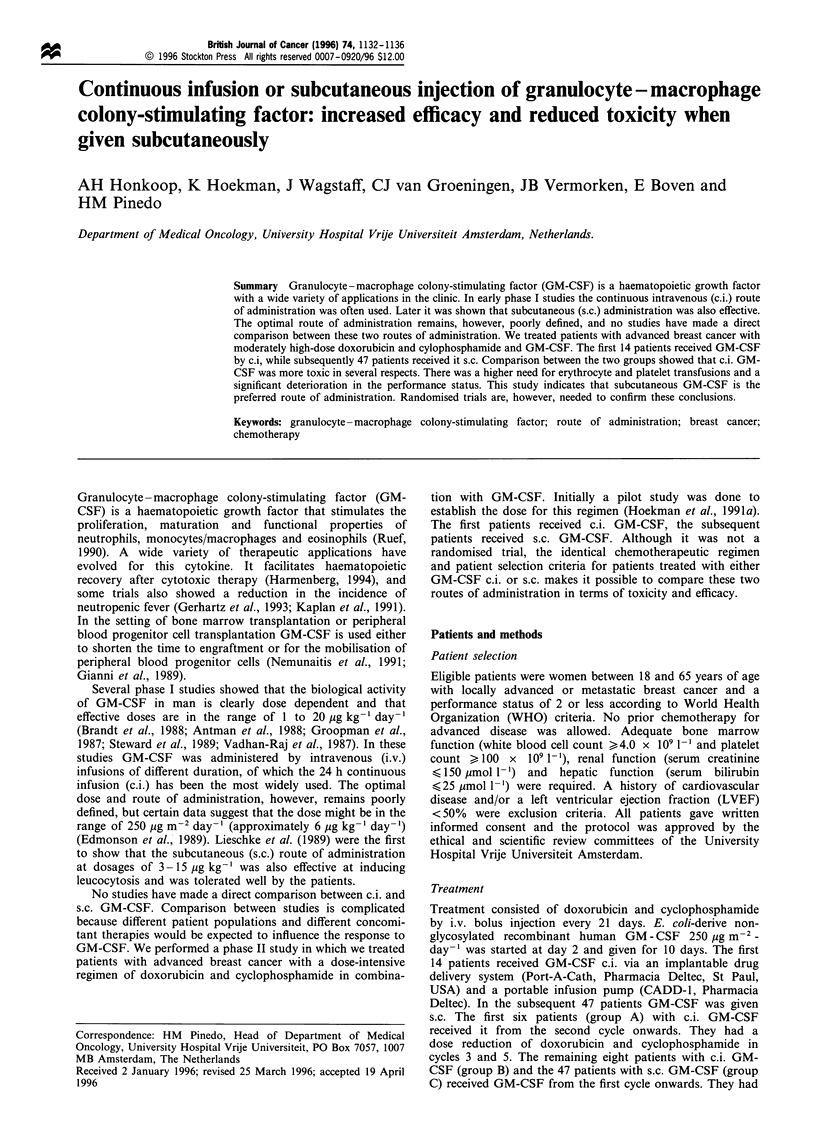

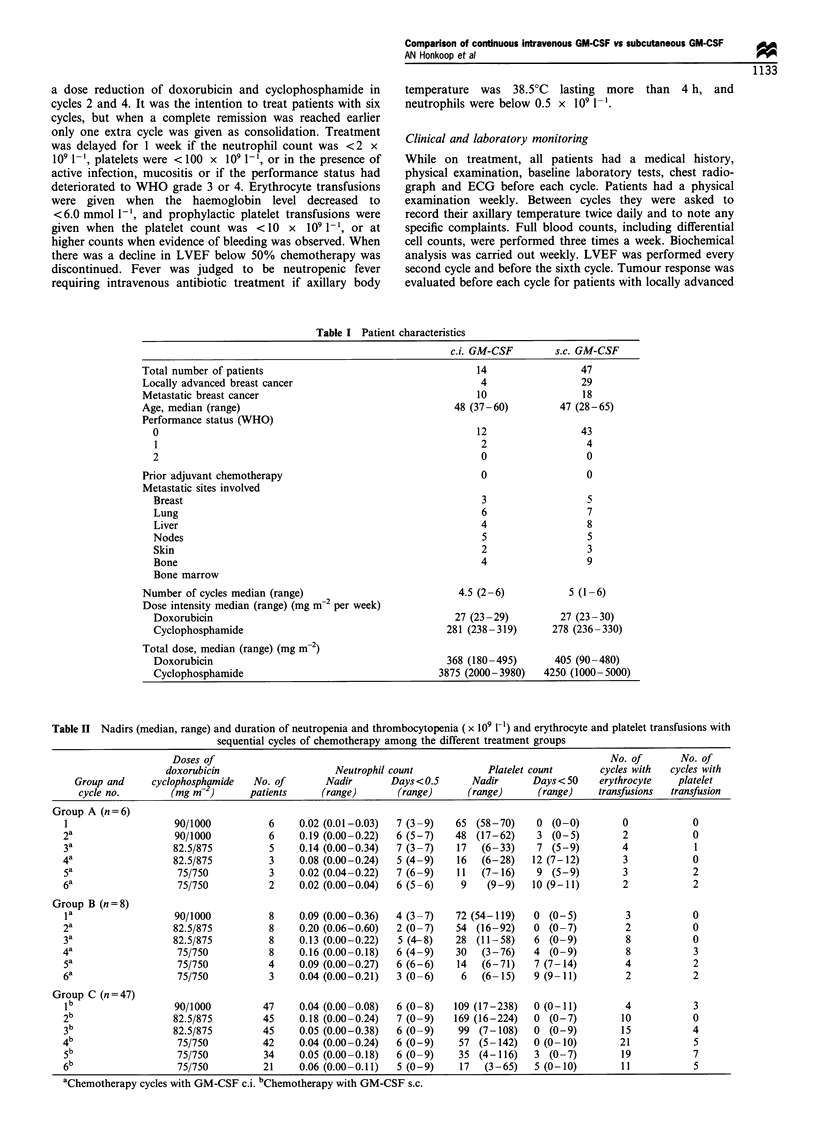

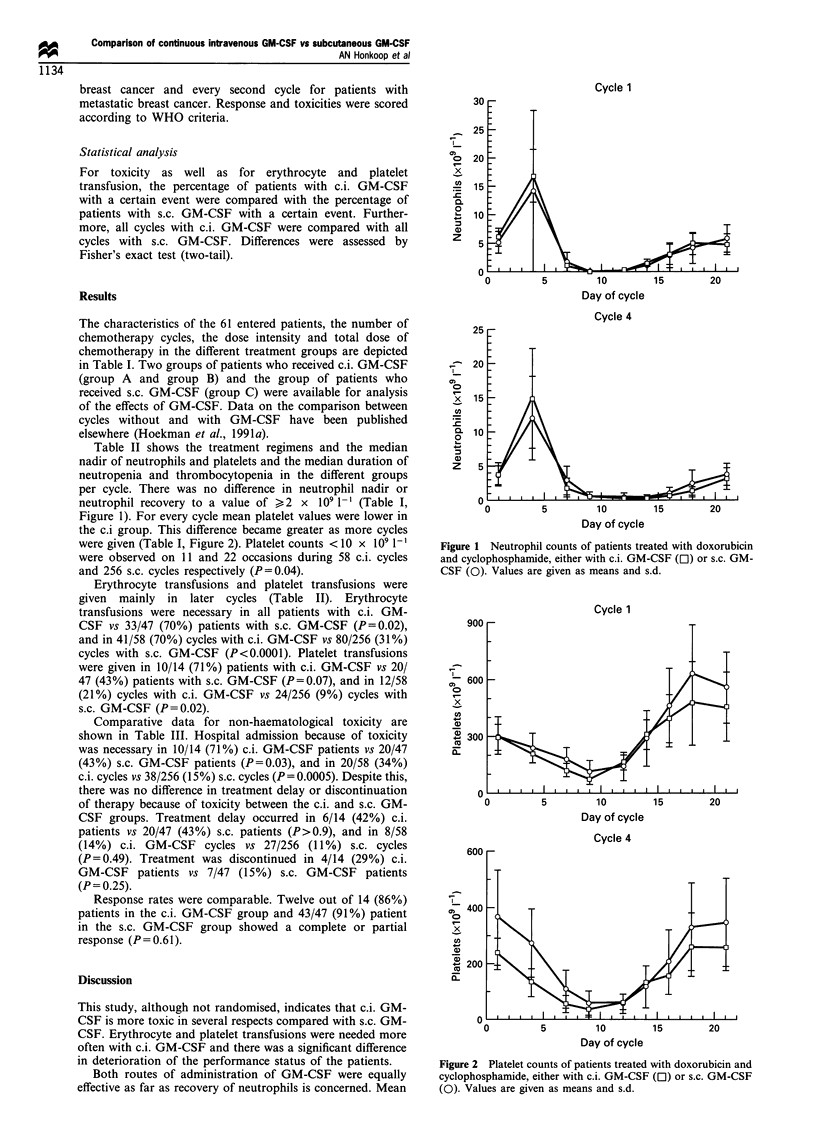

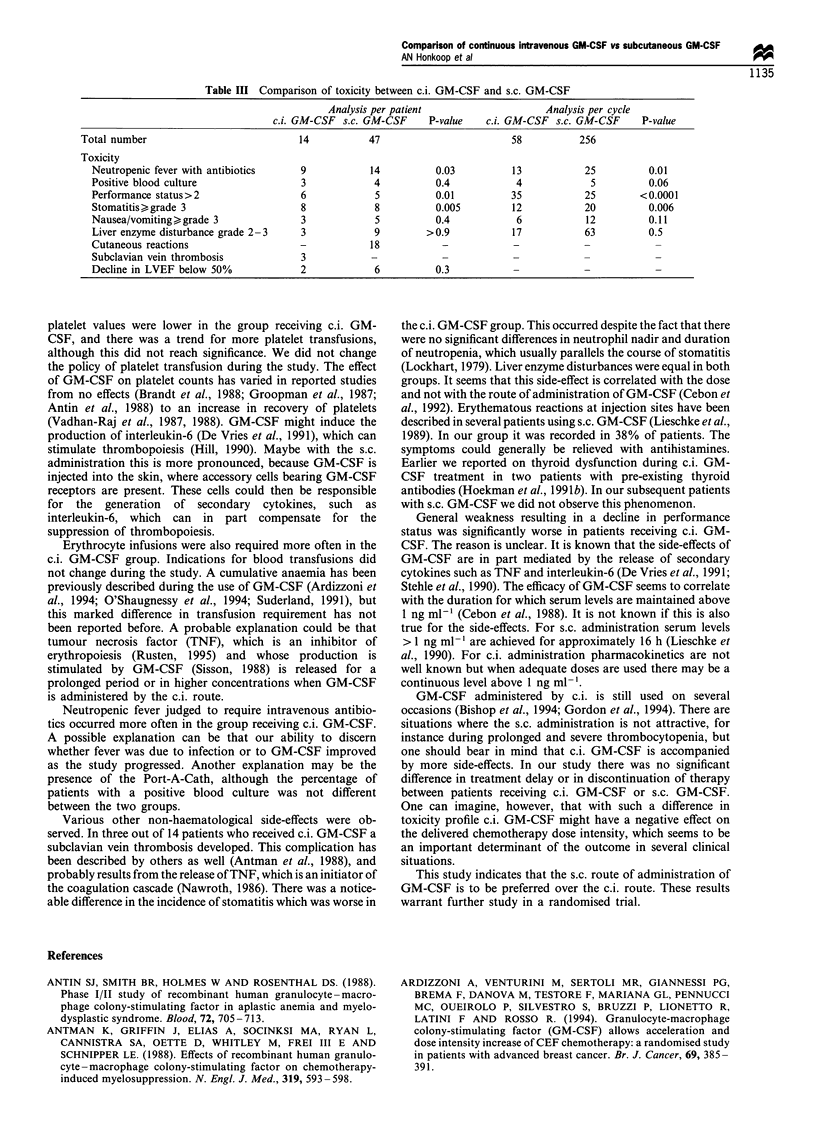

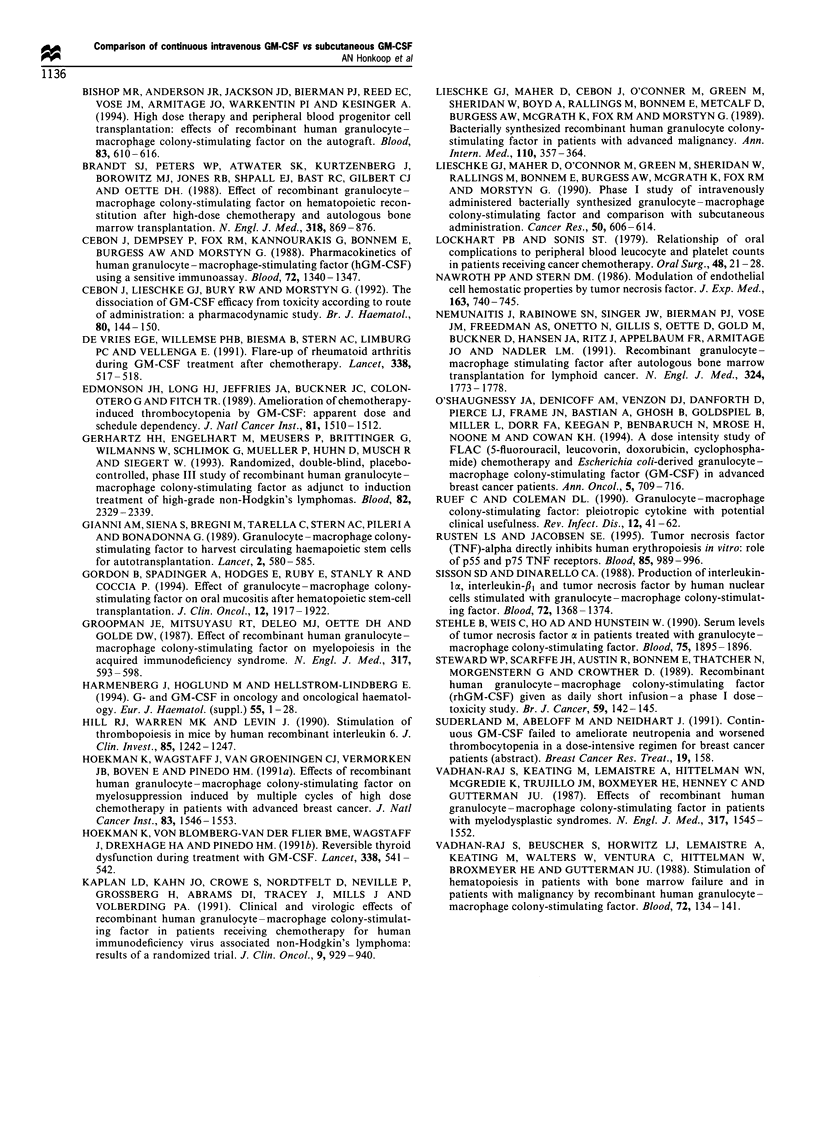


## References

[OCR_00605] Antin J. H., Smith B. R., Holmes W., Rosenthal D. S. (1988). Phase I/II study of recombinant human granulocyte-macrophage colony-stimulating factor in aplastic anemia and myelodysplastic syndrome.. Blood.

[OCR_00612] Antman K. S., Griffin J. D., Elias A., Socinski M. A., Ryan L., Cannistra S. A., Oette D., Whitley M., Frei E., Schnipper L. E. (1988). Effect of recombinant human granulocyte-macrophage colony-stimulating factor on chemotherapy-induced myelosuppression.. N Engl J Med.

[OCR_00619] Ardizzoni A., Venturini M., Sertoli M. R., Giannessi P. G., Brema F., Danova M., Testore F., Mariani G. L., Pennucci M. C., Queirolo P. (1994). Granulocyte-macrophage colony-stimulating factor (GM-CSF) allows acceleration and dose intensity increase of CEF chemotherapy: a randomised study in patients with advanced breast cancer.. Br J Cancer.

[OCR_00631] Bishop M. R., Anderson J. R., Jackson J. D., Bierman P. J., Reed E. C., Vose J. M., Armitage J. O., Warkentin P. I., Kessinger A. (1994). High-dose therapy and peripheral blood progenitor cell transplantation: effects of recombinant human granulocyte-macrophage colony-stimulating factor on the autograft.. Blood.

[OCR_00639] Brandt S. J., Peters W. P., Atwater S. K., Kurtzberg J., Borowitz M. J., Jones R. B., Shpall E. J., Bast R. C., Gilbert C. J., Oette D. H. (1988). Effect of recombinant human granulocyte-macrophage colony-stimulating factor on hematopoietic reconstitution after high-dose chemotherapy and autologous bone marrow transplantation.. N Engl J Med.

[OCR_00649] Cebon J., Dempsey P., Fox R., Kannourakis G., Bonnem E., Burgess A. W., Morstyn G. (1988). Pharmacokinetics of human granulocyte-macrophage colony-stimulating factor using a sensitive immunoassay.. Blood.

[OCR_00655] Cebon J., Lieschke G. J., Bury R. W., Morstyn G. (1992). The dissociation of GM-CSF efficacy from toxicity according to route of administration: a pharmacodynamic study.. Br J Haematol.

[OCR_00668] Edmonson J. H., Long H. J., Jeffries J. A., Buckner J. C., Colon-Otero G., Fitch T. R. (1989). Amelioration of chemotherapy-induced thrombocytopenia by GM-CSF: apparent dose and schedule dependency.. J Natl Cancer Inst.

[OCR_00675] Gerhartz H. H., Engelhard M., Meusers P., Brittinger G., Wilmanns W., Schlimok G., Mueller P., Huhn D., Musch R., Siegert W. (1993). Randomized, double-blind, placebo-controlled, phase III study of recombinant human granulocyte-macrophage colony-stimulating factor as adjunct to induction treatment of high-grade malignant non-Hodgkin's lymphomas.. Blood.

[OCR_00683] Gianni A. M., Siena S., Bregni M., Tarella C., Stern A. C., Pileri A., Bonadonna G. (1989). Granulocyte-macrophage colony-stimulating factor to harvest circulating haemopoietic stem cells for autotransplantation.. Lancet.

[OCR_00686] Gordon B., Spadinger A., Hodges E., Ruby E., Stanley R., Coccia P. (1994). Effect of granulocyte-macrophage colony-stimulating factor on oral mucositis after hematopoietic stem-cell transplantation.. J Clin Oncol.

[OCR_00692] Groopman J. E., Mitsuyasu R. T., DeLeo M. J., Oette D. H., Golde D. W. (1987). Effect of recombinant human granulocyte-macrophage colony-stimulating factor on myelopoiesis in the acquired immunodeficiency syndrome.. N Engl J Med.

[OCR_00699] Harmenberg J., Höglund M., Hellström-Lindberg E. (1994). G- and GM-CSF in oncology and oncological haematology.. Eur J Haematol Suppl.

[OCR_00706] Hill R. J., Warren M. K., Levin J. (1990). Stimulation of thrombopoiesis in mice by human recombinant interleukin 6.. J Clin Invest.

[OCR_00709] Hoekman K., Wagstaff J., van Groeningen C. J., Vermorken J. B., Boven E., Pinedo H. M. (1991). Effects of recombinant human granulocyte-macrophage colony-stimulating factor on myelosuppression induced by multiple cycles of high-dose chemotherapy in patients with advanced breast cancer.. J Natl Cancer Inst.

[OCR_00720] Hoekman K., von Blomberg-van der Flier B. M., Wagstaff J., Drexhage H. A., Pinedo H. M. (1991). Reversible thyroid dysfunction during treatment with GM-CSF.. Lancet.

[OCR_00726] Kaplan L. D., Kahn J. O., Crowe S., Northfelt D., Neville P., Grossberg H., Abrams D. I., Tracey J., Mills J., Volberding P. A. (1991). Clinical and virologic effects of recombinant human granulocyte-macrophage colony-stimulating factor in patients receiving chemotherapy for human immunodeficiency virus-associated non-Hodgkin's lymphoma: results of a randomized trial.. J Clin Oncol.

[OCR_00732] Lieschke G. J., Maher D., Cebon J., O'Connor M., Green M., Sheridan W., Boyd A., Rallings M., Bonnem E., Metcalf D. (1989). Effects of bacterially synthesized recombinant human granulocyte-macrophage colony-stimulating factor in patients with advanced malignancy.. Ann Intern Med.

[OCR_00743] Lieschke G. J., Maher D., O'Connor M., Green M., Sheridan W., Rallings M., Bonnem E., Burgess A. W., McGrath K., Fox R. M. (1990). Phase I study of intravenously administered bacterially synthesized granulocyte-macrophage colony-stimulating factor and comparison with subcutaneous administration.. Cancer Res.

[OCR_00750] Lockhart P. B., Sonis S. T. (1979). Relationship of oral complications to peripheral blood leukocyte and platelet counts in patients receiving cancer chemotherapy.. Oral Surg Oral Med Oral Pathol.

[OCR_00752] Nawroth P. P., Stern D. M. (1986). Modulation of endothelial cell hemostatic properties by tumor necrosis factor.. J Exp Med.

[OCR_00760] Nemunaitis J., Rabinowe S. N., Singer J. W., Bierman P. J., Vose J. M., Freedman A. S., Onetto N., Gillis S., Oette D., Gold M. (1991). Recombinant granulocyte-macrophage colony-stimulating factor after autologous bone marrow transplantation for lymphoid cancer.. N Engl J Med.

[OCR_00769] O'Shaughnessy J. A., Denicoff A. M., Venzon D. J., Danforth D., Pierce L. J., Frame J. N., Bastian A., Ghosh B., Goldspiel B., Miller L. (1994). A dose intensity study of FLAC (5-fluorouracil, leucovorin, doxorubicin, cyclophosphamide) chemotherapy and Escherichia coli-derived granulocyte-macrophage colony-stimulating factor (GM-CSF) in advanced breast cancer patients.. Ann Oncol.

[OCR_00776] Ruef C., Coleman D. L. (1990). Granulocyte-macrophage colony-stimulating factor: pleiotropic cytokine with potential clinical usefulness.. Rev Infect Dis.

[OCR_00781] Rusten L. S., Jacobsen S. E. (1995). Tumor necrosis factor (TNF)-alpha directly inhibits human erythropoiesis in vitro: role of p55 and p75 TNF receptors.. Blood.

[OCR_00788] Sisson S. D., Dinarello C. A. (1988). Production of interleukin-1 alpha, interleukin-1 beta and tumor necrosis factor by human mononuclear cells stimulated with granulocyte-macrophage colony-stimulating factor.. Blood.

[OCR_00794] Stehle B., Weiss C., Ho A. D., Hunstein W. (1990). Serum levels of tumor necrosis factor alpha in patients treated with granulocyte-macrophage colony-stimulating factor.. Blood.

[OCR_00800] Steward W. P., Scarffe J. H., Austin R., Bonnem E., Thatcher N., Morgenstern G., Crowther D. (1989). Recombinant human granulocyte macrophage colony stimulating factor (rhGM-CSF) given as daily short infusions--a phase I dose-toxicity study.. Br J Cancer.

[OCR_00818] Vadhan-Raj S., Buescher S., LeMaistre A., Keating M., Walters R., Ventura C., Hittelman W., Broxmeyer H. E., Gutterman J. U. (1988). Stimulation of hematopoiesis in patients with bone marrow failure and in patients with malignancy by recombinant human granulocyte-macrophage colony-stimulating factor.. Blood.

[OCR_00810] Vadhan-Raj S., Keating M., LeMaistre A., Hittelman W. N., McCredie K., Trujillo J. M., Broxmeyer H. E., Henney C., Gutterman J. U. (1987). Effects of recombinant human granulocyte-macrophage colony-stimulating factor in patients with myelodysplastic syndromes.. N Engl J Med.

[OCR_00662] de Vries E. G., Willemse P. H., Biesma B., Stern A. C., Limburg P. C., Vellenga E. (1991). Flare-up of rheumatoid arthritis during GM-CSF treatment after chemotherapy.. Lancet.

